# Five-step authorship framework to improve transparency in disclosing contributors to industry-sponsored clinical trial publications

**DOI:** 10.1186/s12916-014-0197-z

**Published:** 2014-10-24

**Authors:** Ana Marušić, Darko Hren, Bernadette Mansi, Neil Lineberry, Ananya Bhattacharya, Maureen Garrity, Juli Clark, Thomas Gesell, Susan Glasser, John Gonzalez, Carolyn Hustad, Mary-Margaret Lannon, LaVerne A Mooney, Teresa Peña

**Affiliations:** Department of Research in Biomedicine and Health, University of Split School of Medicine, Šoltanska 2, 21000 Split, Croatia; University of Split School of Humanities and Social Sciences, Split, Croatia; GlaxoSmithKline, King of Prussia, PA USA; Member of MPIP Initiative Steering Committee, Boston, MA USA; Navigant Consulting, Boston, MA USA; Bristol-Myers Squibb, Princeton, NJ USA; Astellas, Northbrook, IL USA; Amgen, Thousand Oaks, CA USA; On behalf of the International Society for Medical Publication Professionals, Briarcliff Manor, NY USA; Janssen Research &Development, LLC, Raritan, NJ USA; AstraZeneca, Alderley Park, UK; Merck & Co, Inc, Whitehouse Station, NJ USA; Takeda, Deerfield, IL USA; Pfizer, New York, NY USA; AstraZeneca, Wilmington, DE USA

**Keywords:** Authorship, Transparency, Clinical trial, MPIP Initiative, Journal editors, Medical writers, Clinical investigators, Publication professionals

## Abstract

Authorship guidelines have established criteria to guide author selection based on significance of contribution and helped to define associated responsibilities and accountabilities for the published findings. However, low awareness, variable interpretation, and inconsistent application of these guidelines can lead to confusion and a lack of transparency when recognizing those who merit authorship. This article describes a research project led by the Medical Publishing Insights and Practices (MPIP) Initiative to identify current challenges when determining authorship for industry-sponsored clinical trials and develop an improved approach to facilitate decision-making when recognizing authors from related publications. A total of 498 clinical investigators, journal editors, publication professionals and medical writers were surveyed to understand better how they would adjudicate challenging, real-world authorship case scenarios, determine the perceived frequency of each scenario and rate their confidence in the responses provided. Multiple rounds of discussions about these results with journal editors, clinical investigators and industry representatives led to the development of key recommendations intended to enhance transparency when determining authorship. These included forming a representative group to establish authorship criteria early in a trial, having all trial contributors agree to these criteria and documenting trial contributions to objectively determine who warrants an invitation to participate in the manuscript development process. The resulting Five-step Authorship Framework is designed to create a more standardized approach when determining authorship for clinical trial publications. Overall, these recommendations aim to facilitate more transparent authorship decisions and help readers better assess the credibility of results and perspectives of the authors for medical research more broadly.

Please see related article: http://www.biomedcentral.com/1741-7015/12/214.

**Electronic supplementary material:**

The online version of this article (doi:10.1186/s12916-014-0197-z) contains supplementary material, which is available to authorized users.

## Introduction

The designation of authorship is essential for published research to be represented by those who provide significant intellectual contribution to its development and execution. More recently, authorship selection has evolved to confer not only the credit for the research but also accountability and responsibility for accuracy and integrity of the work. Yet, making this determination can be especially challenging due to the ambiguous nature of available authorship guidelines and the increasing complexity of clinical trial research [1]. The most common and frequently referenced authorship guidelines in biomedicine are issued by the International Committee of Medical Journal Editors (ICMJE). The goal of the ICMJE criteria is to enhance transparency in authorship disclosure and ultimately to build trust and credibility with the medical literature readership [2,3]. Another authorship model, referred to as contributorship, lists each person’s contributions to the research and manuscript, even for those who are not authors. One or more of these contributors will fulfill the role as guarantors of the paper [4]. Despite implementation of these and other approaches by journals and trial sponsors, concerns over clarity in disclosure of authorship contributions persist, which ultimately can erode credibility in clinical trial research and related publications [5-10]. These authorship issues often arise in situations where established definitions and guidelines are ambiguous, resulting in variations in interpretation and application that can contribute to a lack of transparency [11]. While ICMJE authorship guidance is intentionally broad to accommodate the diversity of scientific research, a more structured authorship framework is needed to improve consistency and clarity in authorship decision-making for clinical trials given the complexity in design and ongoing evolution [12,13].

Improving disclosure of authorship contributions and writing assistance was identified as one of ten recommendations to close the credibility gap in reporting industry-sponsored research by the Medical Publishing Insights and Practices (MPIP) Initiative [14], a collaboration among pharmaceutical companies with representation from the International Society for Medical Publication Professionals (ISMPP) aimed at elevating trust, transparency and integrity in publishing industry-sponsored studies [15]. This article describes a research collaboration between the MPIP Initiative and academic researchers to identify and address challenging authorship case scenarios for industry-sponsored clinical trials. Discussions about these results with journal editors, clinical investigators, academic collaborators and industry representatives allowed the MPIP Initiative to develop a Five-Step Authorship Framework that facilitates a more prospective, transparent and consistent process when determining authorship for industry-sponsored publications.

## Methods

### Creation of authorship scenarios

In early 2012, members of the MPIP Initiative’s Steering Committee collaborated with multiple respondent groups involved in publication of industry-sponsored clinical trials to identify authorship challenges arising from current guidelines. Following in-depth discussions with seven publication professionals (individuals employed or contracted by industry to organize and disseminate scientific and clinical data through peer reviewed publications), three journal editors and three clinical investigators, the MPIP Initiative Steering Committee and its academic collaborators converted these challenging situations into authorship case scenarios for further testing. An additional flow diagram provides a detailed overview of the authorship research project [see Additional file 1].

### Survey development, validation, and analysis

An online survey utilizing these authorship case scenarios was created to collect qualitative data regarding the rationale for adjudication of authorship decisions in individual cases (that is, granting of authorship, acknowledgement, no recognition, other). The survey also asked for confidence level in authorship decisions and perceived frequency of the situation described in the case. Respondents were asked to answer based on their professional experience, as cases did not mention any specific guidelines. At the end of the survey, respondents were also asked about their awareness of and reliance on specific authorship guidelines, as well as the time point during the clinical trial when they would agree on authorship criteria. The full survey [see Additional file 2] was piloted for content and clarity among a convenience sample of five professionals with experience in publishing or editing clinical trials results. The final survey was created after revising the original version based on comments generated during pilot testing.

The survey was sent via an email link (to avoid duplicate answers) to four respondent groups involved in the publication of clinical trial results: clinical investigators involved in industry-sponsored clinical trials (as of September 2012), editors of general or specialty journals who publish results from industry-sponsored clinical trials (contacted directly by the MPIP Initiative, collaborating publishers, or collaborating editors), publication professionals (as defined above) and medical writers employed or contracted by trial sponsors who collaborate with authors in drafting, revising and editing medical information for peer-reviewed journals and congresses. The last two groups were contacted through ISMPP, the American Medical Writers Association (AMWA), the European Medical Writers Association (EMWA) and the MPIP Initiative Steering Committee. Cases were presented randomly to minimize respondent fatigue. From a total of 498 responses, a sample of at least 96 participants per group enabled estimates with a 10% margin of error for the largest respondent group surveyed (clinical investigators). The online survey remained open until all groups surpassed this sample size by at least 10%.

Adjudications of cases, confidence in answers, and authorship practices of respondents were summarized as frequencies. The answers measuring respondents’ confidence and perceived frequency of scenarios were captured on 6 point scales and categorized into three groups: high confidence/frequency (score 1–2), moderate confidence/frequency (score 3–4) and low confidence/frequency (score 5–6). Open-ended explanations for adjudications were analyzed as qualitative data. Answers were summarized and analyzed using a general inductive approach [16]. Participants’ answers related to each case were analyzed and then common themes were identified through initial coding [17]. Overlapping themes were then grouped and the final set of themes developed. Data were coded and analyzed using NVivo 10 (QSR International Pty Ltd., Warrington, UK).

### Consultations with stakeholders

Quantitative survey results were discussed at two separate MPIP-hosted roundtable meetings: 10 U.S.-based editors and 15 MPIP members (New York, USA, December 2012), and five European-based editors, one journal representative, and seven MPIP members (Alderley Park, UK, April 2013). During these roundtables, a facilitator presented quantitative results from the survey-based study. Through an iterative process of discussion and consensus building, key themes were identified. Findings were further refined through analysis of qualitative survey answers and in-depth discussions with six clinical investigators who participated in the online survey and gave permission for follow-up conversations.

## Results

### Survey respondents

Survey respondents (n = 498) included representation from all four target respondent groups: 145 clinical investigators (29% of respondents), 108 journal editors (22%), 132 publication professionals (26%) and 113 medical writers (23%) (Table 1). The survey reached an international audience, from North America (44%) and Europe (39%) to Asia (13%). A majority of respondents had >5 years of industry-sponsored clinical trial experience (82%) and clinical investigators surveyed had published ≥3 papers from that research (63%). Given the various methods used to contact potential respondents and collect survey data, it was not possible to calculate a response rate or determine any differences between respondents and those who did not take the survey.

**Table 1:** **Survey respondent demographics**

**Characteristic**	**Frequency**	**Percent**
**Respondent group:**		
Clinical investigator	145	29.1
Journal editor	108	21.7
Publication professional	132	26.5
Medical writer	113	22.7
**Total**	**498**	**100.0**
**Years associated with industry-sponsored clinical trial:**		
3 to 5 years	89	17.9
6 to 10 years	116	23.3
11 to 20 years	176	35.3
20+ years	117	23.5
**Total**	**498**	**100.0**
**Number of industry-sponsored clinical trial publications as an author in the past 10 years** ^**a**^		
0 to 2 papers	54	37.2
3 to 5 papers	38	26.2
6 to 10 papers	21	14.5
11 to 20 papers	17	11.7
20+ papers	15	10.4
**Total**	**145**	**100.0**

^a^Note: Clinical investigators only.

### Authorship guidelines

**Figure 1:**
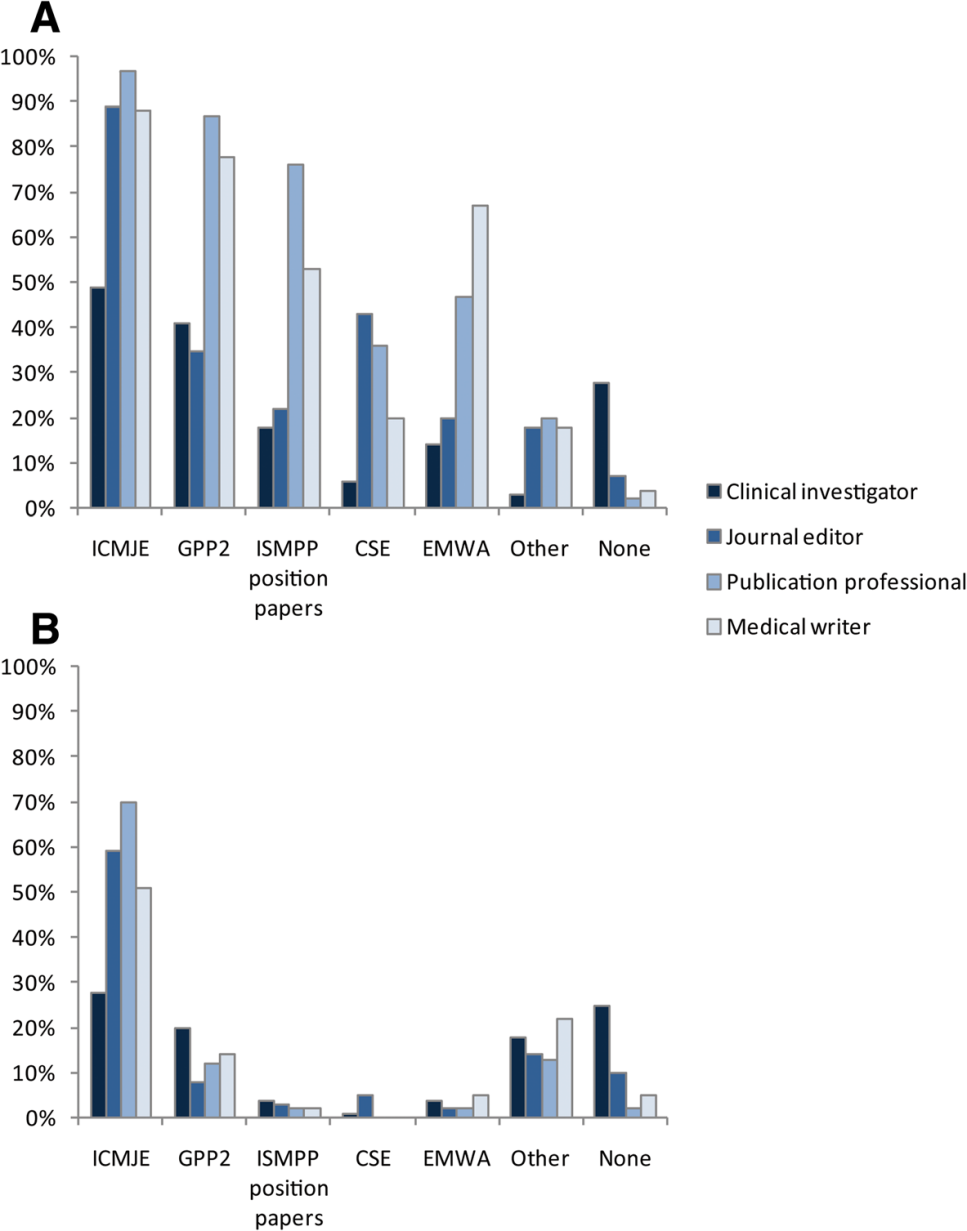
**Respondents’ familiarity (A) with and reliance (B) on authorship guidelines.** More than one guideline could be chosen for familiarity. CSE, Council of Science Editors; EMWA, European Medical Writers Association; GPP2, Good publication practice for communicating company sponsored medical research; ICMJE, International Committee of Medical Journal Editors; ISMPP, International Society of Medical Publication Professionals.

Following completion of the case scenario section, respondents were asked about their familiarity and reliance on guidelines applicable to industry-sponsored clinical trial manuscripts. Respondents were most familiar with ICMJE authorship guidelines (49% to 97%), followed by the Good Publication Practice (GPP2) guidelines [18] (35% to 87%) (Figure 1). Clinical investigators were significantly less familiar with these two guidelines (49% and 41%, respectively) and more often than other groups reported they were not aware of any guidelines (28%). Publication professionals had the highest awareness of ICMJE (97%) and GPP2 (87%) guidelines, mirroring results from previous surveys of this professional group [19]. When asked about reliance on other authorship guidelines in addition to specific journal guidelines, all respondents selected ICMJE as their top choice, although this was less the case among clinical investigators (28%) than other respondent groups (51% to 70%). Clinical investigators reported using no guidelines (25%) more frequently than other groups (2% to 10%) when deciding questions of authorship.

### Authorship case scenario responses

**Figure 2:**
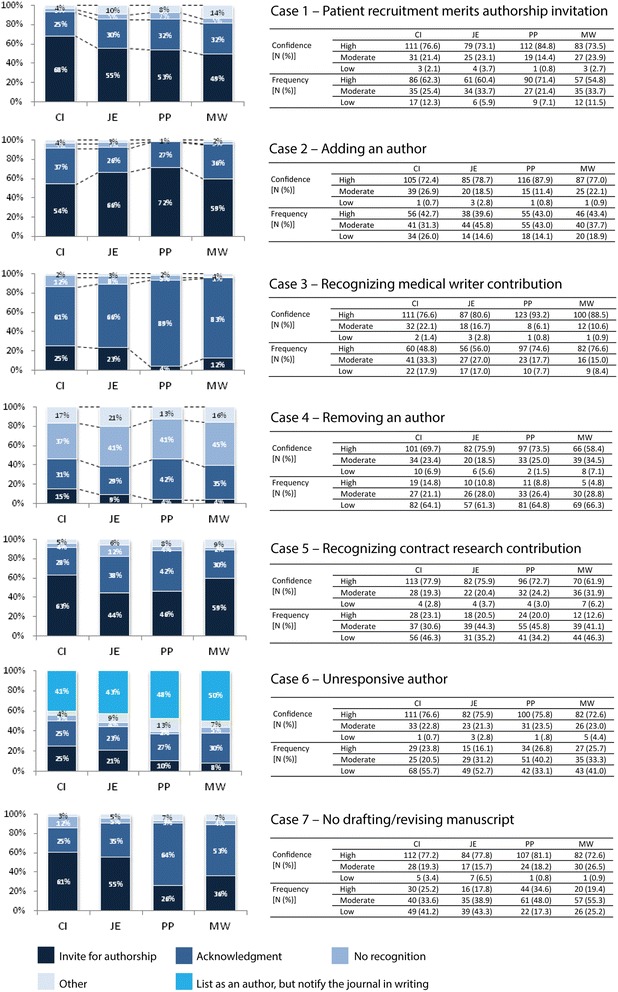
**Case scenario adjudication responses by respondent group.** Abbreviations: CI, Clinical investigator; JE, Journal editor; MW, Medical writer; PP, Publication professional. Confidence in adjudication and perceived frequency of scenarios were captured on 6 point scales and categorized into three categories: high confidence/frequency (score 1-2), moderate confidence/frequency (score 3-4) and low confidence/frequency (score 5-6). "Don't know" responses (<10% for any group) are not included.

Full descriptions of the seven hypothetical authorship case scenarios for industry-sponsored studies and answers by respondent group are presented in Figure 2 and Additional file 3: Table S1. We describe in more detail the cases with greater disagreement among respondent groups and relevance for industry-sponsored clinical trials.

Case 1 tests whether patient recruitment and trial site management were perceived to be significant enough contributions to merit an authorship invitation. Most of the respondents (49% to 68%) agreed that these contributions warranted an invitation to authorship. The rationale for this decision is illustrated by the response from a clinical investigator: ‘Recruiting patients is one of the most important tasks in a clinical trial, and the quality of the study depends on compliance with the inclusion and exclusion criteria. Therefore, this is probably one of the most important persons for the study, and should therefore take responsibility/be acknowledged as an author.’ This case was estimated by respondents to occur most frequently, and >73% of respondents in each group were confident in their answer. ICMJE criteria for authorship available at the time of the survey [20] were unclear as to whether patient recruitment was considered to be a substantial contribution as part of data acquisition.

Case 2 explores the two issues: 1) whether a statistician who is believed to have made a substantial contribution but did not contribute to trial design or drafting of the manuscript merits an invitation to authorship; and 2) whether timing of authorship criteria fulfillment impacts the decision. More publication professionals supported consideration for authorship (72%) compared to clinical investigators (54%). Qualitative responses highlighted a lack of consensus between respondent groups. One editor commented that ‘Authorship on a manuscript needed to be decided before the writing of a first draft’, in contrast to a clinical investigator who wrote ‘Authorship should reflect a significant contribution to the substance of the study - it was an error not to include this person from the start.’ While respondents had generally high confidence in their answers (>72% for all groups), they reported moderate frequency for this case. ICMJE criteria in place at the time of the survey [20] provided guidance on authorship criteria but did not address the issue of timing for the fulfillment of the criteria.

Case 3 asks if a contribution from a medical writer could qualify the person as an author. In this case, a medical writer is hired to help draft the manuscript from a trial report and supports authors in manuscript development under the guidance of the authors to final acceptance. Nearly one quarter of journal editors (23%) and clinical investigators (25%) supported authorship in this case. Respondents were aware this choice was not concordant with guidelines, exemplified by a clinical investigator: ‘I believe medical writers should be listed as authors in those instances where they have contributed from the first draft through to submission. (I recognize that this would require revision of ICMJE guidelines).’ This case was estimated by respondents to be the second most frequent, and >76% of respondents in each group were confident in their answer. ICMJE guidelines at the time of survey [20] stated that writing assistance, technical editing, language editing, and proofreading did not qualify as a substantial contribution.

Finally, Case 7 tests whether providing those who make a significant contribution the opportunity to serve as an author takes precedence over protection of proprietary information. In this scenario, respondents were asked if a clinician who leaves a trial sponsor company for a competitor after making a substantial contribution but prior to drafting the manuscript should be invited to contribute as an author. A majority of clinical investigators (61%) and journal editors (55%) were in favor of inviting the clinician to serve as an author versus publication professionals (26%) and medical writers (36%). As with other cases, respondents were more often concerned about what was fair and not what was prescribed, as noted by a publication professional: ‘Honestly speaking, I think the clinician should be recognized as an author, but he does not meet authorship criteria.’ More than 72% of respondents in each group were confident in their answer. This case was estimated to occur relatively infrequently.

When considering answers from the cases as a whole, three overarching insights emerged. First, responses pertaining to granting authorship, acknowledgement, no recognition or other, varied not only across but within the four respondent groups for each case. This variability suggests limited agreement within peer groups as to how to determine authorship on challenging cases, as well as differences in opinion between the respondent groups. Second, a majority of respondents in all groups were confident in their answers regardless of the case subject, answer choice or perceived frequency. Finally, analysis of 2,841 out of 3,486 possible open-ended answers (81% response rate) highlighted key justifications underlying authorship decisions in the survey (Table 2). These themes included the importance or significance of the contribution for each scenario, alignment or misalignment with formal authorship guideline criteria, importance of transparency and/or responsibility and need for prior agreement on authorship criteria [see Additional file 4 for example answers]. The group of respondents who would grant authorship in case scenarios more often used the ‘importance of contributions’ in explaining their adjudication (57%) versus those who would grant acknowledgment or not offer authorship. The latter respondents most often cited the following rationales: ‘importance of contribution’ (30%), application of ‘formal criteria’ (38%) and ‘transparency/responsibility’ (26%).

**Table 2:** **Themes used in adjudication answers when invitation for authorship was chosen versus all other answers**

	**Number of references coded within the theme (% of all coded references within the adjudication choice/choices)**	
**Theme**	**Grant authorship**	**Acknowledgement/No authorship/Other**
Importance of contribution	608 (57%)	381 (30%)
Formal criteria	277 (26%)	478 (38%)
Transparency/Responsibility	149 (14%)	319 (26%)
Agreement in advance	30 (3%)	69 (6%)

As clinical investigators with two or fewer published articles from clinical trials constitute approximately a third of the respondent population (Table 1), we compared case adjudications from this group with that from other respondents. This sensitivity analysis revealed a difference only for Case 1, where relatively inexperienced clinical investigators would be less likely to invite a clinical investigator who enrolled the most patients to help draft/revise the manuscript. There were no differences in confidence or perceived frequency of the case scenarios, suggesting that publication experience had minimal influence on the answers provided.

### Consultations with editors

The quantitative survey results from the case scenarios were shared with fifteen U.S. and five European-based journal editors and one journal representative across two separate discussion-based roundtables to gain further insights and begin developing recommendations. Feedback highlighted the following areas to help improve authorship disclosure:

- Authorship criteria should be determined as early as possible in the course of a clinical trial;
- Authorship criteria can vary between clinical trials, assuming current guidelines are followed;
- Authorship criteria should be agreed to by all trial contributors via authorship agreements;
- Trial contributions should be documented and summarized prior to publication; and,
- The entire author list should agree to any authorship changes during the publication process.

Quantitative survey results and these preliminary themes were subsequently discussed with six clinical investigators from our survey sample via in-depth teleconference interviews to help further refine initial recommendations.

### Five-step authorship framework

Using input from quantitative survey results, analysis of qualitative open-ended answers, and discussions with journal editors, clinical investigators and industry representatives, key themes were identified that led to the development of the following ‘Five-Step Authorship Framework to Improve Transparency in Disclosing Contributors to Industry-sponsored Clinical Trial Publications (Table 3).

**Table 3:** **Five-Step Authorship Framework to Improve Transparency in Disclosing Contributors to Industry-sponsored Clinical Trial Publications**

**Step**	**Tasks**
**1**	Establish an authorship working group of core trial contributors as close as possible to trial commencement but prior to enrollment of first patient. For smaller trials where an authorship working group is not feasible, this committee may be a sub-committee of a larger Trial Steering Committee where one exists.
**2**	Determine, in the context of the ICMJE authorship criteria and the specific trial, which authorship contributions will qualify as ‘substantial’ and include these details in written publication agreements for all trial contributors.
**3**	Implement a process to track and document in a concise format progress toward substantial contributions based on agreed-upon qualifications.
**4**	Assess documented trial contributions for the specific study and invite those making a ‘substantial contribution’ to participate as author(s).
**5**	Ensure invited authors meet remaining ICMJE authorship criteria during content development and publication submission.

#### Step 1 – Establish an authorship working group early in the trial

This working group should include core trial contributors with broad representation from multiple disciplines, including the trial Steering Committee, clinical investigators with relevant expertise, study co-sponsors and external experts. Where possible, these working group members should remain unchanged throughout the study to help ensure continuity. Members of the working group need not be authors nor should they be guaranteed authorship without meeting authorship criteria for the trial.

#### Step 2 – Determine substantial contribution criteria

The primary task for the authorship working group is to determine prospectively which contributions will qualify as ‘substantial’ in the context of trial activities. These criteria should align with external guidelines (for example, ICMJE, GPP2 and journals being considered for submission) and policies of the sponsor, and they should be finalized prior to first patient enrollment. Standardized language for those criteria should be included in written publication agreements and agreed to by all trial contributors, including those in the authorship working group.

When determining these authorship criteria, trial activities meriting an invitation for authorship should be evaluated by their ability to impact broader trial outcomes compared to those serving a more narrow function. It is important to note that definitions for substantial contributions can differ between trials, provided external guidelines such as ICMJE are followed, since trial activities vary greatly across therapeutic areas, stages of development, sponsors and other dimensions. When considering large, multi-national trials, it is especially important to clearly determine substantial contribution criteria as early as possible in the trial, given the large number of contributors involved.

#### Step 3 – Document trial contributions

In addition to setting the criteria for substantial contribution, the authorship working group should create a process for tracking and documenting all relevant trial contributions that meet the pre-defined criteria as substantial. This process should be made transparent in the written publication agreement, and trial contributors should be notified of the requirement to provide this information as part of ongoing trial responsibilities. To avoid creating new tasks and expense, tracking should be included among normal trial activities where possible.

#### Step 4 – Determine those making a substantial contribution

Once trial activities for a given manuscript are completed, the authorship working group should reconvene to apply the criteria to the documented contributions. All trial contributors, regardless of function, responsibility or relationship to trial sponsor(s), meeting the authorship criteria for substantial contribution determined in Step 2 should then be invited to draft/revise the manuscript. However, an invitation for authorship may be declined by a contributor. As part of our discussions with journal editors and clinical investigators, it was noted that while up-front participation is preferred, later contributions sometimes rise to the level of a substantial contribution. The authorship working group should have the responsibility to determine which potential authors made a substantial contribution based on the contribution data and adjudicate disagreements that arise from its decisions. Contributions which do not meet the standard for ‘substantial’ should be agreed to by all authors and noted in the acknowledgements.

#### Step 5 – Ensure authors meet remaining authorship criteria

Those who accept an invitation to draft/revise the manuscript will serve as the initial author list. The final responsibility of the authorship working group is to ensure timely dissemination of findings that are consistent with the pre-specified aims of the clinical trial. Those in the author list should agree on their role prior to drafting the manuscript and all contributions should be fully documented. It is important that members of the author list fulfill the remaining authorship criteria beyond substantial contribution outlined in the written publication agreement. Changes to the author list can occur, either adding those who make a later substantial contribution or removing those who do not fulfill all authorship requirements. However, to maintain transparency, all authorship changes and the accompanying rationale should be documented and agreed to by the entire author list. At submission, the authors should consider providing a summary of the author list and their specific contributions to indicate how authorship was determined.

## Discussion

Given the growing demand for increased transparency in publications for clinical trials [21-24], the MPIP Initiative sought to identify authorship ambiguities encountered in industry-sponsored trials not well addressed by current guidelines and to develop an approach by which authorship decisions can be more clearly and easily determined. Investigation of the rationale used by the respondent groups in adjudicating challenging case scenarios identified a number of key gaps between authorship guidelines and how these scenarios are addressed. Most important among these, survey results highlighted the variability in responses both across and within peer groups despite some groups having high familiarity with and reliance on current authorship guidelines. Some cases also tested a number of areas where authorship criteria do not explicitly provide guidance, such as whether patient recruitment is considered a substantial contribution or the impact of timing on authorship criteria fulfillment [20], which further contributed to response variability. These findings suggest the lack of clear authorship guidance on some scenarios frequently encountered in real life, and indicate that respondents acting on judgment contrary to guidance can contribute to lack of transparency. Second, despite this heterogeneity, respondents were uniformly confident in their own rationale for these authorship decisions. Since the survey did not specify or refer to any guidelines during the case scenario section, respondents likely believed their experience and judgment are a correct approach to resolving these scenarios. Finally, clinical investigators, who make up a significant percentage of authors on manuscripts from clinical trials, appear to be concerned with the importance or significance of the contribution rather than following external guidelines to determine authorship. This perspective is exemplified by clinical investigators ranking lowest among the groups surveyed for awareness of authorship criteria.

Through discussions with journal editors, clinical investigators and industry representatives on the gaps in authorship decision-making that emerged from the survey, we identified key themes for improvement. These themes included defining the criteria for substantial contributions early in the trial, ensuring all trial contributors agree to these criteria and documenting contributions to ensure these criteria are met. Expanding on these key insights, the Five-step Authorship Framework was developed to provide a clear and flexible process to facilitate more transparent and consistent authorship decision-making.

The success of the Five-Step Authorship Framework relies significantly on convening a working group early in the trial process to discuss which trial activities should be considered a substantial contribution in the context of current authorship guidelines. Given the wide diversity of trials undertaken and likely future changes in authorship guidelines, defining a comprehensive set of potential activities that meet the definition of ‘substantial’ for authorship is neither practical nor durable. Instead, utilizing the experience and judgment of a diverse group of core trial contributors in an open discussion format provides a transparent process for determining fair and comprehensive authorship criteria for a given trial. In addition, these authorship criteria should align with external guidelines, such as ICMJE and GPP2, to avoid potential bias in selection of criteria that exclude legitimate authorship. Documenting these definitions in authorship agreements ensures that all trial contributors are informed in advance and provides a basis for conflict resolution should a dispute arise. The flexibility of this framework permits authorship criteria specific to each trial, as some trial groups may define significant patient recruitment to be a substantial contribution while others may not (see Case 1). This adaptable format will help facilitate the continued movement away from patient recruitment as sole criteria for substantial contribution and place greater emphasis on intellectual contributions, thus better aligning with the evolving concept of authorship.

The authorship working group should also be responsible for determining who has fulfilled the criteria of substantial contribution to help draft the manuscript, ensuring the results are consistent with the aims of the trial. The initial author list should be determined through matching the documented contributions of potential pre-specified authors to the authorship criteria. In this way, the authorship working group would have a data-driven approach to determine which potential authors fulfill criteria 1 of the ICMJE authorship recommendations (see Cases 3, 5 to 7). In addition, the pre-specified author criteria provide an objective basis for evaluating whether to add or remove an author as contributions come to light during manuscript development (see Cases 2 and 4). Given the importance of its responsibilities, the authorship working group should be a stand-alone group and not subsumed into the trial Steering Committee. As outlined, the authorship working group will not require substantial additional burden and, hence, convening such a group should be feasible.

Our survey results identified divergent opinions both within and across respondent groups for how to adjudicate the cases, with varying awareness and reliance on authorship guidelines. Previous research has shown that authorship issues can be seen as either matters of defined rules or obligation [25]. Decisions about issues using defined rules may differ by time or place, whereas for obligation, higher order rules define the right and wrong courses for action, and decisions are not contingent on specific social rules [26]. Our research confirms that authorship decisions bring forth issues both based on rules (‘formal criteria’) and obligation (‘importance of contributions’), and participants try to compromise between these perspectives. Our finding that trial publication experience has little influence on the scenario adjudication suggests these opinions are not generated by clinical trial experience but are rather a characteristic inherent to the professionals taking part in clinical trials. Therefore, forming a diverse group early in the trial can facilitate this process by bringing together those who are more aware of guidelines and setting mutually agreed upon authorship criteria from both domains through broad discussion. These findings also independently align with the recent change in ICMJE authorship requirements, which added accountability for all aspects of work described in the manuscript as the fourth criterion [2].

Our study has limitations related to its methodological approach, which was focused on collecting qualitative input from professional groups involved in industry-sponsored clinical trial publications rather than quantitative data, which can be difficult to collect via online surveys [27,28]. The survey contained the possibility for self-selection bias, with respondents potentially having a special interest in authorship and, therefore, not being representative. However, the survey was not meant to collect representative input across all relevant characteristics from the four respondent groups but to learn about possible approaches to solving real-life authorship challenges in clinical research to inform potential solutions to these problems. In addition, the Five-step Authorship Framework will not resolve all authorship questions, especially as clinical trials continue to evolve in complexity. We see the Framework as a living guideline that will change together with clinical research.

The companies included in this publication will support implementation of this tool in their clinical trials to supplement best practice and would encourage other industry members to do so as well to ensure this framework is broadly embedded for maximum impact. Given the contribution from other stakeholders, such as journal editors, the authors will be reaching out to them and other organizations to ask for their help to foster awareness and uptake for the Five-step Framework.

## Conclusions

This research outlines an approach for manuscripts from industry-sponsored clinical trials that emphasizes early determination of substantial contribution by a diverse group of core participants, clear communication of authorship criteria to all participants and documentation of contributions that leads to less opaque authorship decisions. This Five-Step Authorship Framework has the potential to facilitate decision-making with improved transparency and consistency when recognizing authors for all clinical trial publications, not just those from industry-sponsored efforts.

## Additional files

Additional file 1: Figure S1. Time line for the MPIP Authorship Research project with inclusion of principal steps in step-wise fashion. Additional file 2: Survey S1. Full online authorship survey. Additional file 3: Table S1. Case scenarios used in the survey. Additional file 4: Table S2. Representative examples of qualitative responses by respondent group and case categorized by different themes.

## Abbreviations

CI: clinical investigator
CSE: Council of Science Editors
EMWA: European Medical Writers Association
GPP2: Good publication practice for communicating company sponsored medical research
ICMJE: International Committee of Medical Journal Editors
ISMPP: International Society of Medical Publication Professionals
JE: journal editor
MPIP Initiative: Medical Publishing Insights and Practices Initiative
MW: medical writer
PP: publication professional
