# Supplementary material for: Five-step authorship framework to improve transparency in disclosing contributors to industry-sponsored clinical trial publications
Source: BMC Med. 2014 Oct 24;12:197. doi: 10.1186/s12916-014-0197-z (PMC4209055; doi:10.1186/s12916-014-0197-z)
Supplement: Additional file 1: Figure S1. — Time line for the MPIP Authorship Research project with inclusion of principal steps in step-wise fashion. [file 12916_2014_197_MOESM1_ESM.docx]

Additional file 1: Figure S1. Timeline for the MPIP Authorship Research project with inclusion of principal steps in step-wise fashion.

Development of authorship case scenarios

Analysis of qualitative survey feedback to determine rationale and key themes for quantitative answers

2 meetings and in-depth interview discussions of quantitative survey results to develop draft recommendations

Analysis of quantitative survey results

Distribution of online authorship survey

Finalized MPIP Five-Step Authorship Framework
