# Supplementary material for: Five-step authorship framework to improve transparency in disclosing contributors to industry-sponsored clinical trial publications
Source: BMC Med. 2014 Oct 24;12:197. doi: 10.1186/s12916-014-0197-z (PMC4209055; doi:10.1186/s12916-014-0197-z)
Supplement: Additional file 2: Survey S1. — Full online authorship survey. [file 12916_2014_197_MOESM2_ESM.docx]

Additional file 2: Survey S1. MPIP Authorship Survey

**MPIP Authorship Survey**

September 17^th^, 2012

*[New screen]*

**Introduction**


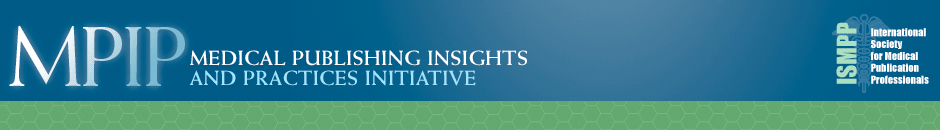


Thank you for taking the time to fill out the following online survey from the Medical Publishing Insights and Practices (MPIP) initiative.

The goal of this survey is to explore how key stakeholders**resolve authorship questions forindustry-sponsored clinical trial publications**. Individual responses will be kept **strictly confidential**, but the overall results will be published and used to inform development of recommendations to guide authorship decisions when questions arise in the future.

If you would like to learn more about the results of the study, please enter your email here (optional):

_______________________________________

*[New screen]*

**Background**

1. Please choose which classification best describes your **currentprofessional affiliation**. (Check all that apply)
   1. **Clinical investigator** involved with industry-sponsored clinical trials
   2. **Editor** of a general or specialty journal that publishes results from industry-sponsored clinical trials
   3. **Publication professional*** who has experience with publication of clinical trial results either at a pharmaceutical/biotech company or a medical communications company
   4. **Medical writer**** who is contracted or employed by pharmaceutical/biotech to assist in drafting and/or revising manuscripts resulting from clinical trials
   5. **Other**; Please specify _____________________**-> TERMINATE**

*Publication professional – involved in the planning and dissemination of scientific and clinical data related to drug development through peer reviewed publications, scientific congresses, and other venues.

**Medical writer –involved in drafting, revising, and editing medical content for peer reviewed journals, regulatory agencies, and other medical audiences.

*[New screen]*

1. **In whichcountry** have you acquired the majority of your professional experiencewith industry-sponsored clinical trials?

*[Note: Insert drop down list of countries]*

*[New screen]*

1. **Over the course of your entire career**, howmany years have you been involved or associated with industry-sponsored clinical trials?
   1. 0-2 years**-> TERMINATE, otherwise continue**
   2. 3-5 years
   3. 6-10 years
   4. 11-20 years
   5. 20+ years

**[NOTE: Ask Q4 if Q1 answer is a]**

*[New screen]*

1. For how many industry-sponsored clinical trial publications have you **appeared as an author within the past 10 years**?
   1. 0-2 papers
   2. 3-5 papers
   3. 6-10 papers
   4. 11-20 papers
   5. 20+ papers

*[New screen]*

Please read carefully the following**7 hypothetical authorship scenarios** that are based on real world experiences whererecognition of individual contributions to industry-sponsored clinical trials is in question. Based on your professional experience, please indicate**how you wouldrecognize these contributions** should you be asked to provide a suggestion.

*[New screen]*

**[NOTE: Cases will be randomly selected to rotate order]**

**[NOTE: Percentage of survey completed will be shown on the bottom part of each screen]**

**Case 1**

A clinical investigatorinvolved with an industry-sponsored clinical trial enrolled the most patients from dozens of investigators. This clinical investigatordid notcontribute to the trial design, butwas involved with the day-to-day management of the trial at their institution. This clinical investigator feels the number of recruited patients and daily trial management duties were substantial and this contribution merits an invitation for authorship on the manuscript.

1. In your opinion, what would be the **most appropriate way to recognize the contribution** of the investigator in question?
   1. I would invite the investigator to help draft the manuscript as an author listed in the byline
   2. I would list the investigator’s contribution in the acknowledgement section
   3. I would not invite the investigator to be an author nor recognize the investigator in the manuscript
   4. Other; Please specify _____________________
2. Please explain your rationale for your decision above.*[Free response]*

*[Note: Answers to the next two questions will appear as scales, with radio buttons corresponding to the numbers. Only the extreme ends of the scale will be labeled]*

1. Please rate your **level of confidence** in your decision regarding how to recognize the individual’s contribution.

|  |  |  |  |  |  |
| --- | --- | --- | --- | --- | --- |
| Extremely confident |  |  |  |  | Not at all confident |

1. In your view, how **frequently**do you think this scenario occurs in clinical trial reporting?

|  |  |  |  |  |  |  |
| --- | --- | --- | --- | --- | --- | --- |
| Extremely frequent |  |  |  |  | Not frequent | Don't know |

*[New screen]*

**[Note: Ask Q9 if Q5 answer is other than a]**

1. In your opinion, what would the clinical investigator from this caseneed to do in order to beconsidered for an invitation for authorship?*[Free response]*

*[New screen]*

**Case 2**

The trial team of a large multicenter clinical studycompleted a first draft of a manuscript and began revisions. The lead clinical investigatorwants to extend an invitation for authorshipto a statistician, who is part of the trial team from the company sponsoring the clinical study.The lead clinical investigator believes the statisticiancontributed substantially todata analysis and interpretation. The trial team notes this statistician has not participated in trial design or drafting of the manuscript to date.

1. Inyour opinion, what would be the **most appropriate way torecognize the contribution** of the statistician?
   1. I would invite the statistician to critically review and revise the manuscript and be listed as an author in the byline
   2. I would list the statistician’s contribution in the acknowledgement section
   3. I would not invite the statistician to be an authornor recognize the statistician in the manuscript
   4. Other; Please specify _____________________
2. Please explain your **rationale** for your decision above.*[Free response]*

*[Note: Answers to the next two questions will appear as scales, with radio buttons corresponding to the numbers. Only the extreme ends of the scale will be labeled]*

1. Please rate your **level of confidence** in your decision regarding how to recognize the individual’s contribution.

|  |  |  |  |  |  |
| --- | --- | --- | --- | --- | --- |
| Extremely Confident |  |  |  |  | Not at all confident |

1. In your view, how **frequently**do you think this scenario occurs in clinical trial reporting?

|  |  |  |  |  |  |  |
| --- | --- | --- | --- | --- | --- | --- |
| Extremely frequent |  |  |  |  | Not frequent | Don't know |

*[New screen]*

**[Note: Ask Q14 if Q10 answer is other than a]**

1. In your opinion, what would the statistician need to do in order to be considered for authorship?*[Free response]*

*[New screen]*

**Case 3**

The trial team for an industry-sponsored clinical studycompleted an internal trial report. They then hired a medical writer to draft theinitial outline and manuscript based on the internal trial report. The medical writer continued to provide support in manuscript development through the revision and submission process until a final version of the manuscript was acceptedfor publication in a peer reviewed journal.

1. Inyour opinion, what would be the **most appropriate way to recognize the contribution** of the medical writer?
   1. I would invite themedical writer to be an author listed in the byline
   2. I would listthe medical writer’s contribution in the acknowledgement section
   3. I would not invite the medical writer to be an authornor recognize the medical writer in the manuscript
   4. Other; Please specify _____________________
2. Please explain your **rationale** for your decision above.*[Free response]*

*[Note: Answers to the next two questions will appear as scales, with radio buttons corresponding to the numbers. Only the extreme ends of the scale will be labeled]*

1. Please rate your **level of confidence** in your decision regarding how to recognize the individual’s contribution.

|  |  |  |  |  |  |
| --- | --- | --- | --- | --- | --- |
| Extremely confident |  |  |  |  | Not at all  confident |

1. In your view, how **frequently** do you think this scenario occurs in clinical trial reporting?

|  |  |  |  |  |  |  |
| --- | --- | --- | --- | --- | --- | --- |
| Extremely frequent |  |  |  |  | Notfrequent | Don't know |

*[New screen]*

**[Note: Ask Q19 if Q15 answer is other than a]**

1. Are there situations where a **medical writer** for a primary manuscript from an industry-sponsored clinical trial **should be invited to be an author**?
   1. Yes;Please specify _____________________
   2. No

*[New screen]*

**Case 4**

A clinical investigatormadesubstantial contributions to trial design, data analysis, and manuscript revisions, and is listed as an author on a manuscriptprior to submission. At a late stage of revising the manuscript, the clinical investigator expresses disagreement with the data interpretation and conclusions as statedin the manuscript and does not want to be listed as an authoror be acknowledged in the publication.

For the purpose of this case, assume this clinical investigator represents a dissenting view despite rigorous and robust scientific debate. Also assume there are no ethical or integrity issues with the study conduct or data reporting, this is purely a difference of scientific interpretation.

1. Inyour opinion, what would be the **mostappropriate way to recognize the contribution** of the clinical investigator?
   1. I would continue to list the investigator as an author in the byline
   2. I would remove the investigator from the byline but list the investigator in the acknowledgement section
   3. I would not recognize the investigator in the manuscript
   4. Other; Please specify _____________________
2. Please explain your **rationale** for your decision above.*[Free response]*

*[Note: Answers to the next two questions will appear as scales, with radio buttons corresponding to the numbers. Only the extreme ends of the scale will be labeled]*

1. Please rate your **level of confidence** in your decision regarding how to recognize the individual’s contribution.

|  |  |  |  |  |  |
| --- | --- | --- | --- | --- | --- |
| Extremely confident |  |  |  |  | Not at all  confident |

1. In your view, how **frequently**do you think this scenario occurs in clinical trial reporting?

|  |  |  |  |  |  |  |
| --- | --- | --- | --- | --- | --- | --- |
| Extremelyfrequent |  |  |  |  | Not frequent | Don't know |

*[New screen]*

**Case 5**

A contract research organization (CRO)was hired to perform biomarker analysis for an industry-sponsored clinical trial. A scientist at the CRO developed aproprietary biomarker assay that was essential for the trial. Prior to drafting of the manuscript, the scientistsuggeststhe novel nature and integral role of theassay represents a substantial contribution to the researchand, thus, the scientist feels justified in requesting authorship on the manuscript.

1. Inyour opinion, what would be the **most appropriate way to recognize the contribution** of the CRO scientist?
   1. I would invite the CRO scientist to help draft the manuscript as an author listed in the byline
   2. I would recognize the CRO scientist’s contribution in the acknowledgement section
   3. I would not recognize the CRO scientist in the manuscript
   4. Other; Please specify _____________________
2. Please explain your **rationale** for your decision above.*[Free response]*

*[Note: Answers to the next two questions will appear as scales, with radio buttons corresponding to the numbers. Only the extreme ends of the scale will be labeled]*

1. Please rate your **level of confidence** in your decision regarding how to recognize the individual’s contribution.

|  |  |  |  |  |  |
| --- | --- | --- | --- | --- | --- |
| Extremely confident |  |  |  |  | Not at all  confident |

1. In your view, how **frequently** would an analogous situation from a CRO or similar contracted organization occur?

|  |  |  |  |  |  |  |
| --- | --- | --- | --- | --- | --- | --- |
| Extremely frequent |  |  |  |  | Not frequent | Don't know |

*[New screen]*

**Case 6**

A clinical investigator contributed substantially to the trial design, data interpretation, drafting, and revision of several drafts of the manuscript. Prior to the first manuscript submission, the lead author made multiple attempts to contact this clinical investigator to obtain final approval for the manuscript without receiving any response.

1. Inyour opinion, what would be the **most appropriate way to recognize the contribution** of the unresponsive clinical investigator?
   1. I would continue to list the investigator as an author in the byline
   2. I would remove the investigator from the byline and list in the acknowledgement section
   3. I would not recognize the investigator in the manuscript
   4. I would list the investigator as an author but explain in the cover letter to the journal that it had not been possible to receive formal approval of the submitted manuscript version
   5. Other; Please specify _____________________
2. Please explain your **rationale** for your decision above. *[Free response]*

*[Note: Answers to the next three questions will appear as scales, with radio buttons corresponding to the numbers. Only the extreme ends of the scale will be labeled]*

1. Please rate your **level of confidence** in your decision regarding how to recognize the individual’s contribution.

|  |  |  |  |  |  |
| --- | --- | --- | --- | --- | --- |
| Extremelyconfident |  |  |  |  | Not at all  confident |

1. **How important** is it to have **final approval** from the unresponsive investigator **prior to the first manuscript submission**?

|  |  |  |  |  |  |
| --- | --- | --- | --- | --- | --- |
| Extremely important |  |  |  |  | Not at all  important |

1. In your view, how **frequently** do you think this scenario occurs in clinical trial reporting?

|  |  |  |  |  |  |  |
| --- | --- | --- | --- | --- | --- | --- |
| Extremely frequent |  |  |  |  | Not frequent | Don't know |

*[New screen]*

**Case 7**

A clinician, who substantially contributed to trial design, data analysis, and data interpretation,leaves the company sponsoring the trialfor a new position witha competitor companybefore the manuscript is drafted.The company sponsoring the trial does not allow the clinician to take part in drafting the manuscript, to prevent access to what is now perceived as“proprietary information.” The clinician argues to be invited to serve as an author based on past contributions and the central role played in the trial.

1. Inyour experience, what would be the **most appropriate way to recognize the contribution** of the clinician?
   1. I would include the clinician as an author in the byline
   2. I would recognize the clinician’s contribution in the acknowledgement section
   3. I would not recognize this clinician in the publication
   4. Other; Please specify _____________________
2. Please explain your **rationale** for your decision above.*[Free response]*

*[Note: Answers to the next three questions will appear as scales, with radio buttons corresponding to the numbers. Only the extreme ends of the scale will be labeled]*

1. Please rate your **level of confidence** in your decision regarding how to recognize the individual’s contribution.

|  |  |  |  |  |  |
| --- | --- | --- | --- | --- | --- |
| Extremely confident |  |  |  |  | Not at all  confident |

1. Based on the specified contribution listed above, **how important is drafting of the manuscript** to the decision on who should be invited as an author?

|  |  |  |  |  |  |
| --- | --- | --- | --- | --- | --- |
| Extremely important |  |  |  |  | Not at all  important |

1. In your view, how **frequently** do you think this scenario occurs in clinical trial reporting?

|  |  |  |  |  |  |  |
| --- | --- | --- | --- | --- | --- | --- |
| Extremely frequent |  |  |  |  | Not frequent | Don't know |

*[New screen]*

**Authorship Practices**

1. With which of these**external guidelines**are you **familiar**? Please check all that apply.
   1. International Committee of Medical Journal Editors (ICMJE)
   2. Good Publication Practice (the GPP2 guidelines)
   3. International Society for Medical Publication Professionals (ISMPP) position papers
   4. Council of Science Editors (CSE)
   5. European Medical Writers Association (EMWA) guidelines
   6. Other; Please specify _____________________
   7. None
2. In addition to the guidelines of specific journals to which you may be submitting a manuscript, which of these guidelines do you **rely onmost** when deciding questions of authorship?
   1. International Committee of Medical Journal Editors (ICMJE)
   2. Good Publication Practice (the GPP2 guidelines)
   3. International Society for Medical Publication Professionals (ISMPP) position papers
   4. Council of Science Editors (CSE)
   5. European Medical Writers Association (EMWA)
   6. Institutional / Company guidelines and policies
   7. Other; Please specify _____________________
   8. None
3. In your experience, for a given clinical study when do you typically**decide thecriteria** to determine authorship?
   1. During trial protocol development
   2. At trial completion but prior to manuscript development
   3. At first draft of manuscript
   4. During revision of the manuscript
   5. After completion of manuscript but prior to submission
   6. Other; Please specify _____________________
4. When in your experience do you typically determine**whowill be invited** to serve as an author?
   1. During trial protocol development
   2. At trial completion but prior to manuscript development
   3. At first draft of manuscript
   4. During revision of the manuscript
   5. After completion of manuscript but prior to submission
   6. Other; Please specify _____________________
5. What**model** should form the **basis for recognizing contributions** to industry-sponsored clinical trial research?
   1. Authorship model*
   2. Contributorship model**
   3. Other; Please specify _____________________

*An Authorship Model lists a set of authors on a byline who as a group accept responsibility for the manuscript content (e.g., ICMJE).

**A Contributorship Modelhas each author describe their work during the course of the study from inception through publication and designate their functional role within the group.
