# Supplementary material for: Five-step authorship framework to improve transparency in disclosing contributors to industry-sponsored clinical trial publications
Source: BMC Med. 2014 Oct 24;12:197. doi: 10.1186/s12916-014-0197-z (PMC4209055; doi:10.1186/s12916-014-0197-z)
Supplement: Additional file 3: Table S1. — Case scenarios used in the survey. [file 12916_2014_197_MOESM3_ESM.docx]

**Table S1.** Case scenarios used in the survey

| **Case 1 – Patient recruitment merits authorship invitation.** A clinical investigator involved with an industry-sponsored clinical trial enrolled the most patients from dozens of investigators. This clinical investigator did not contribute to the trial design, but was involved with the day-to-day management of the trial at their institution. This clinical investigator feels the number of recruited patients and daily trial management duties were substantial and this contribution merits an invitation for authorship on the manuscript. |
| --- |
| **Case 2 – Adding an author.** The trial team of a large multicenter clinical trial completed a first draft of a manuscript and began revisions. The lead clinical investigator wants to extend an invitation for authorship to a statistician who is part of the trial team from the company sponsoring the trial. The lead clinical investigator believes the statistician contributed substantially to data analysis and interpretation. The trial team notes this statistician has not participated in trial design or drafting of the manuscript to date. |
| **Case 3 – Recognizing medical writer contribution.** The trial team for an industry-sponsored clinical study completed a trial report. They then hired a medical writer to help draft the initial manuscript based on the trial report. The medical writer continued to provide support in manuscript development through the revision and submission process until a final version of the manuscript was accepted for publication. |
| **Case 4 – Removing an author.** A clinical investigator made substantial contributions to trial design, data analysis, and manuscript revisions, and is listed as an author on a manuscript prior to submission. At a late stage of revising the manuscript, the clinical investigator expresses disagreement with the data interpretation and conclusions as stated in the manuscript and does not want to be listed as an author or be acknowledged in the publication.; For the purpose of this case, assume this clinical investigator represents a dissenting view despite rigorous and robust scientific debate. Also assume there are no ethical or integrity issues with the study conduct or data reporting. |
| **Case 5 – Recognizing contract research contribution.** A contract research organization (CRO) was hired to perform biomarker analysis for a clinical trial. A scientist at the CRO developed a proprietary biomarker assay that was essential for patient segmentation in the trial. Prior to drafting of the manuscript, the scientist suggests the novel nature and integral role of the biomarker assay represents a substantial contribution to the research and, thus, the scientist feels justified in requesting authorship on the manuscript. |
| **Case 6 – Unresponsive author.** A clinical investigator contributed substantially to the trial design, data interpretation, drafting, and revision of the manuscript. Prior to the first manuscript submission, the lead author made multiple attempts to contact this clinical investigator to obtain final approval for the manuscript without receiving any response. |
| **Case 7 – No drafting/revising manuscript.** A clinician, who substantially contributed to trial design, data analysis, and data interpretation, leaves the company sponsoring the trial for a competitor before the manuscript is drafted. The company sponsoring the trial does not allow the clinician to take part in drafting the manuscript, to prevent access to what is now perceived to be “proprietary information.” The clinician argues to be invited to serve as an author based on past contributions and the central role played in the trial. |
