# Supplementary material for: Five-step authorship framework to improve transparency in disclosing contributors to industry-sponsored clinical trial publications
Source: BMC Med. 2014 Oct 24;12:197. doi: 10.1186/s12916-014-0197-z (PMC4209055; doi:10.1186/s12916-014-0197-z)
Supplement: Additional file 4: Table S2. — Representative examples of qualitative responses by respondent group and case categorized by different themes. [file 12916_2014_197_MOESM4_ESM.docx]

Additionalfile 4: Table S2. Representative examples of qualitative responses by respondent group and case categorized by different themes*

| **Theme**  (Total number of references coded within the theme) | **Case**  (Total number of references coded within the theme) | **Examples** |
| --- | --- | --- |
| **Agreement in advance** (n=99) | **Case 1 –**Patient recruitment merits authorship invitation (n=25) | - *But this really depends on what was agreed on in advance, and how flexible this agreement was. If there was an advance agreement saying no authorship and changing that would upset other investigators, then acknowledge the person.* (Editor) - *These issues need to be addressed prior to the implementation of the study and it should clearly state what are the terms of authorship and acknowledgement.*(Editor) - *This would depend in part on prespecified authorship criteria: these should have prespecified recognition dependent on contribution.*(Clinical investigator) - *I would have aimed to establish how authors would be determined early in the trial.* (Publication professional) - *Authorship should only be reserved to those involved in the trial design, unless decided differently from the beginning.*(Medical writer) |
|  | **Case 2 –** Adding an Author (n=20) | - *Authorship on a manuscript needed to be decided before the writing of a first draft.* (Editor) - *Authorship should reflect a significant contribution to the substance of the study. It was an error not to include this person from the start.*(Clinical investigator) - *The manuscript is already under development and authorship should be decided before drafting of the manuscript occurs.*(Publication professional) - *This is not an ideal situation and highlights the importance of confirming all authors during the author kick-off call.* (Publication professional) - *Was not involved from the beginning - authorship should be decided at the outset.* (Medical writer) |
|  | **Case 3 –** Acknowledging a Medical Writer (n=6) | - *Depends on the pre-hiring agreement with the writer.* (Editor) - *It depends on the agreement that was established with the medical writer at the time of contracting them.* (Clinical investigator) - *This is challenging. And it should NOT be made as a retrospective decision, but agreed upon upfront... to me this would depend on how the project was set up and how the project progressed.*(Publication professional) - *As the medical writer was hired for drafting the trial report, he/she should not be included as author. Anyway, at the time of hiring, these issues are addressed and clarified in the hiring agreement.* (Publication professional) - *The work was advanced and the medical writer joined the workstream too late in the process to be considered for an authorship invitation.* (Medical writer) |
|  | **Case 4 –** Removing an Author (n=3)  [No responses from journal editors and clinical investigators] | - *It's is choice not to agree with data interpretation but he/she should have expressed this disagreement earlier.*(Publication professional) - *Ideally this situation would be avoided by adequate upfront discussions with all authors about the data and their interpretation.*(Medical writer) - *These answers are too black and white. Everything should be done to get the author on board prior to submission. Given his/her contributions, it is simply not good enough that the author is removed entirely.*(Medical writer) |
|  | **Case 5 –** Contribution from contracted organization (n=16) | - *Typically, the rights of the CRO to publish or not to be allowed to publish the work is specified in a research contract prior to the work being carried out.*(Editor) - *If this is being agreed upon in advance of the data and clinical trial being conducted it would make sense.*(Clinical investigator) - *I am presuming that a part of the CRO agreement was the agreement that whilst under employment, any proprietary discoveries belonging to the place of employment.* (Publication professional) - *Actually, this scenario should be regarded in advance in a treaty between CRO and industry. In providing support as a CRO usually the CRO looses [sic] any proprietary rights.*(Medical writer) |
|  | **Case 6 –** No/Unclear Final Approval (n=7)  [No responses from journal editors and clinical investigators] | - *An author agreement should have been used in the beginning that set expectations for authorship.*(Publication professional) - *It in part depends on how the project was set up and expectations, eg, sometimes the lead author has been given discretion in such matters.*(Publication professional) - *Final approval is part of the authorship criteria, and the investigator (hopefully) was advised of that from the beginning. If the investigator does not respond to requests for final approval, he has not fulfilled authorship criteria.* (Medical writer) - *I would let the author know in advance that we would have to remove him/her if we do not receive his final author approval.*(Medical writer) |
|  | **Case 7–**No drafting/revising of manuscript (n=22) | - *They should have made a written agreement on what will happen to the data; without it the clinician has waived his or her right on this manuscript authorship.* (Editor) - *Contribution is acknowledged. Authorship is refused as he/she has terminated the contract with the sponsor, unless specified otherwise in the contract.* (Editor) - *If at the beginning of the project, this clinician was promised authorship and fulfilled his/her roles, authorship should be granted.*(Clinical investigator) - *These situations are usually covered in an employment contract with the clinician. If allowed and acceptable to the sponsoring company, this individual could be recognized in the acknowledgements.*(Publication professional) - *Authorship should only be reserved to those involved in the trial design, unless stated differently from the beginning.*(Medical writer) |
| **Transparency/ Responsibility**  (n=468) | **Case 1 –**No/Unclear Substantial Contribution (n=50) | - *Being "rewarded" for number of subjects is a conflict of interest.*(Editor) - *PIs are usually professionals who influence population of enrolled patients, during trial and in follow up period gather real life experience with the drug and form their professional impression and opinion about trial compound that can significantly contribute discussion and conclusions of publication. This is often more objective then conclusions driven only by statistical data analysis by authors contracted by company.* (Clinical investigator) - *I would list the investigator’s contribution in the acknowledgement section along with ALL other physicians who enrolled patients; mentioning the trial site/hospital. Enrolling physicians could be ordered on basis of number of recruited patients.*(Publication professional) - *May have substantial patient treatment experience that could be valuable input to a manuscript.* (Publication professional) - *If the investigator collected data for the study and then contributed to the writing of the manuscript and provided final approval, he/she would meet authorship criteria. However, this scenario depends on whether there is a limit on the number of authors allowed by the target journal and if other persons are more qualified to be authors.* (Medical writer) |
|  | **Case 2 –** Adding an Author (n=40) | - *Recognition of the statistician is particularly important when he/she is paid directly by the study's sponsor. Authorship improves transparency.* (Editor) - *In an industry sponsored trial, getting post-hoc creative with statistics is not appropriate.*(Editor) - *The statistician must participate in the writing of the manuscript to receive authorship, and they need to be able to take responsibility for its contents.*(Clinical investigator) - *The contribution of the Statistician should be recognized; however, it should be clearly mentioned that the statistician was not involved in trial design/drafting of manuscript and that he/she was only contributed in the data analysis and interpretation.*(Publication professional) - *Statisticians often know the data better than anyone else, and are in the best position to justify the analyses that we conducted.* (Medical writer) |
|  | **Case 3 –** Acknowledging a Medical Writer (n=85) | - *Transparency is required for all industry trials and "ghost writers" need to be acknowledged but they would not normally be listed as authors because of lack of involvement in study design.*(Editor) - *As a matter of transparency, the medical writer must be disclosed and his contribution acknowledged.* (Clinical investigator) - *It is the medicals writer’s job to write the manuscript. He/she should be acknowledged in the manuscript. If there are errors the medical writer needs to take responsibility. Placing the authors name gives the person responsibility for work done.* (Clinical investigator) - *The writer must be acknowledged, at minimum, to clearly communicate the medical writer's role. If the team felt the work was worthy of authorship, I would not be against addition to the byline.* (Publication planer) - *If the medical writer contributed scientifically, then s/he should be invited for authorship with an explicit statement that the medical writer did not get involved in any part of study conduct and only contributed after the study ended.* (Medical writer) |
|  | **Case 4 –** Removing an Author (n=172) | - *This disagreement needs to be indicated in the publication of this article, so that the reader will know the participants and also the fact that one of the key involved individuals disagreed with the final result.*(Editor) - *It would not be appropriate to simply remove the author and pretend the disagreement doesn't exist. If the author was not prepared to remain on the paper with a split discussion then they should be removed from the paper but the alternative interpretation should still be recognized in the discussion.*(Clinical investigator) - *The investigator's autonomy should be respected. The Editor should be informed and may wish to invite the dissenting investigator to comment on the paper at the same as it is published.* (Clinical investigator) - *I would want to comply with the wishes of the Investigator however on submission of the manuscript I would make the position transparent to the journal editor and seek his/her advice. Usually such issues would be resolved through issue resolution meetings.*(Publication professional) - *Being an author implies legal responsibility for the content of the paper. If the author does not want to be listed as an author or in the acknowledgements, they should not be mentioned.*(Medical writer) |
|  | **Case 5 –** Contribution from contracted organization (n=21) | - *I believe that transparency around the involvement of the CRO scientist as an author is the best way forward.* (Editor) - *Authors are responsible for content, the development of the assay is part of the content.* (Clinical investigator) - *While he might not be able to defend the trial as a whole, his contribution was essential and potentially only he can defend one key aspect of the trial - the accuracy and reliability of the biosassay.* (Publication professional) - *[Authorship should be given] Only if that scientist took full responsibility for the reporting of that data in the paper.* (Publication professional) - *Because the biomarker assay is proprietary, the scientist can't provide enough details in the paper for a reader to run the assay so the scientist should be acknowledged but not be an author.* (Medical writer) |
|  | **Case 6 –** No/Unclear Final Approval (n=69) | - *It is important to have full disclosure. In fact, if the manuscript were to be accepted for publication and the author did not respond, that should be made clear in the published article.* (Editor) - *It is important for all members to accept the manuscript and disclosure of inability to contact is ethical.*(Clinical investigator) - *Investigator has fulfilled most criteria for authorship. Inappropriate simply to list as author as final criterion is lacking. Explaining to journal editor details of situation is the transparent and honest approach.*(Publication professional) - *Transparency between the submitting author and the journal editor is the path I would choose; a request from a journal editor to the clinical investigator may return a response from the clinical investigator.* (Publication professional) - *To be listed as an author, the contributor needs to accept responsibility for its content. One cannot assume that. Therefore, they cannot be listed as an author.*(Medical writer) |
|  | **Case 7–** No drafting/revising of manuscript (n=31) | - *If the original company considers the information proprietary, how can readers of the journal be confident that all the important information is included in the manuscript?*(Editor) - *The author is being excluded due to circumstances outside of the scientific or editorial merit of the work, and rather this appears to be a political or economic situation. As a journal author, I feel that I have a role to play in ensuring the transparency of authorship in publications.* (Editor) - *The work to be published will be the clinician's work and if any individuals are to be identified then this person needs to be, for transparency.*(Clinical investigator) - *Although the clinician did not fulfill ALL of the requirements for authorship (i.e., no involvement in the ms) in the interest of full disclosure this individual should be recognized for their contributions.*(Publication professional) - *The clinician played a substantial role and the manuscript should be transparent about that by recognizing their contribution in the acknowledgement section.* (Medical writer) |
| **Formal criteria** (n=755) | **Case 1 –**No/Unclear Substantial Contribution (n=116) | - *Again, just because you have accrued a large number of patients to a clinical trial, does not necessitate authorship as defined by ICMJE.*(Editor) - *This is a major contribution to the conduct of the study and is equivalent to the data from a major experiment being used in a laboratory based manuscript. Again, involvement of this investigator in drafting the manuscript then has the potential to fulfill the other criteria for authorship.*(Clinical investigator) - *The investigator did not meet ICMJE authorship criteria. Further, ICMJE specifically states that patient recruitment is not sufficient for authorship.*(Medical writer) - *ICMJE guidelines are the authorship guidelines recommended by GPP2 to follow for authorship suggestions. Criteria 1 says clearly someone should have participated in study design in order to be considered as an author.*(Publication professional) - *The contributions do not satisfy authorship but the investigator should be given the opportunity to participate as an author if they are willing to make the effort.* (Publication professional) |
|  | **Case 2 –** Adding an Author (n=119) | - *If they haven't drafted or critically revised the manuscript then they don't meet the ICMJE criteria for authorship.* (Editor) - *If statistician contributed substantially to data analysis and interpretation then should be an author, if also reviews & revises manuscript.*(Clinical investigator) - *Substantial contribution that does not qualify for authorship in Acknowledgments.* (Medical writer) - *Statisticians who substantially contribute to data analysis and interpretation as well as critical review and revision of manuscript fulfill ICMJE criteria for authorship.* (Publication professional) - *If the statistician's contribution to the data interpretation is needed for the manuscript then she can address her parts and offer critical review of the rest of the manuscript. I believe this follows ICMJE criteria.* (Publication professional) |
|  | **Case 3 –** Acknowledging a Medical Writer (n=126) | - *According to my journal's criteria, anyone who writes the initial draft must be included as an author in the byline. We believe whoever writes the first draft sets the framework and sets the tone for all future revisions; therefore, this criterion by itself requires the medical writer to be listed in the author byline. Because we require all authors to state their role in an "Author Contributions" section of the manuscript, the statement in that section will specify the medical writer's role in drafting the outline and manuscript and involvement in revisions and submission.*(Editor) - *The medical writer did not fulfill all the criteria for authorship as per ICMJE criteria. Drafting and writing the manuscript in itself does not qualify for authorship.* (Clinical investigator) - *The medical writer does not meet authorship criteria. However, he/she has made a substantial contribution to the final manuscript. Moreover, his/her contribution has been funded by the sponsor and this should be also recognized.* (Publication professional) - *If the medical writer provides significant intellectual contribution and fulfills ICMJE criteria he/she should be entitled to authorship.* (Medical writer) - *The writer does not meet ICMJE author criteria, but should be acknowledged as per GPP2 guidelines.* (Medical writer) |
|  | **Case 4 –** Removing an Author (n=83) | - *The investigator does not agree with the final version of the manuscript; therefore, he/she does not fulfill the third ICMJE criteria, and thus, he/she is not an author. He/she does not agree with being named in the Acknowledgements section. Since his/her approval for being named in the Acknowledgements section is necessary, we cannot name him/her in this section too.*(Editor) - *Authorship includes approval of the final manuscript to be published. Anything short of this (in this case disagreement with the discussion and conclusions) does not meet ICMJE authorship criteria (although they may qualify for contributorship).* (Editor) - *However, the significant contributions to design, data analysis and revisions merit acknowledgement.* - *He/she didn't agree hence can’t be the author, for all authors have to agree.*(Clinical investigator) - *This person has clearly stated that they do not approve the final version and therefore do not fulfill ICMJE criteria.*(Publication professional) - *An investigator must agree to be an author or acknowledged. If he/she does not agree, he/she cannot be recognized.*(Medical writer) |
|  | **Case 5 –** Contribution from contracted organization (n=77) | - *Collecting data or contributing to laboratory tests alone is not sufficient to meet the ICMJE authorship criteria.*(Editor) - *The person does not fulfill the Vancouver criteria.* (Clinical investigator) - *I would consider the assay as an essential contribution to the trial and thus I believe if fulfills the 1st requirement for authorship. Drafting of the manuscript and approval fulfill the final two.*(Publication professional) - *Author input is essential in this situation. The fact that the scientist works for a CRO should not determine whether the scientist should be included in the process to meet authorship criteria.* (Publication professional) - *The exact criteria for authorship in this circumstance may well depend on journal guidelines; however, given the scientists' contribution, if they were to become more involved in the analysis and drafting, their role would become less ambiguous.*(Medical writer) |
|  | **Case 6 –** No/Unclear Final Approval (n=120) | - *Awkward situation, but approval is needed before submission. This author apparently fulfills requirements for authorship, so deleting him from the byline is not an option.*(Editor) - *Journals require individual authors to sign off on their role and contribution to the manuscript.*(Clinical investigator) - *Under my organizations rules, I must have the final author approval and conflict of interest information to be included in a byline, unless the author pre-deceases submission.*(Publication professional) - *Providing approval for the manuscript submission is one of the requirements for authorship.*(Medical writer) - *I have listed my answer based on ICMJE criteria, however, this can cause issues in the real world.* (Medical writer) |
|  | **Case 7 –** No drafting/revising of manuscript (n=114) | - *According to my journal's criteria, the author, by contributing to the trial design and data analysis and interpretation, has qualified for authorship. There is no need for him to be involved in the writing. However, any person who has been involved in drafting the manuscript also needs to be in the byline according to our criteria, whether or not he has taken part in the study design or data analysis.*(Editor) - *Although it sounds as though this clinician would have earned authorship, there are now legal obstacles to allowing him to fulfill the requirement of contributing to the manuscript for authorship.*(Clinical investigator) - *As long as the clinician has an opportunity to review and comment on the paper, the other contributions are authorship-worthy.* (Clinical investigator) - *Honestly speaking, I think the clinician should be recognized as an author, but he does not meet authorship criteria.*(Publication professional) - *There might be a case of the clinician to be an author but it all depends on whether he/she can fully contribute during the manuscript process and provide final approval, thereby meeting ICMJE authorship criteria.* (Medical writer) |
| **Importance of contribution** (n=989) | **Case 1 –**No/Unclear Substantial Contribution (n=157) | - *Recruiting patients is one of the most important tasks in a clinical trial, and the quality of the study depends on compliance with the inclusion and exclusion criteria. Therefore, this is probably one of the most important persons for the study, and should therefore take responsibility/be acknowledged as an author.*(Editor) - *If the trial would have been delayed/not completed had the investigator not done their part, then it is a significant contribution and should be recognized.*(Clinical investigator) - *Not only did the investigator enroll the most patients, the investigator was actively involved in day-to-day operations and did not delegate all responsibilities.* (Clinical investigator) - *Apart from the trial design the clinical investigator was very much involved with the study and if he agrees to help draft and revise manuscript then he should be considered for authorship.*(Publication professional) - *This author has made a substantial contribution to the acquisition of data, and therefore qualifies as an author (ICMJE) if he/she reviews the manuscript and approves the final version. Investigators who enrolled fewer patients can be listed in the acknowledgements section.*(Medical writer) |
|  | **Case 2 –** Adding an Author (n=191) | - *Statisticians do not create new data, they merely process what others collect. Most of them do not qualify as professional authors with understanding of issues in the specific professional field. Statisticians should be paid for their efforts and acknowledged, but not listed in the bylines.*(Editor) - *Interpretation of statistical analysis of data is a significant contribution that deserves credit in authorship. Statistical analysis without interpretation does not necessarily merit authorship.* (Editor) - *Do you value a good statistician? If you do, you will ask him/her to prepare the stats section and critically review the manuscript and tables for accuracy. If you hate your statistician, you won't.*(Clinical investigator) - *Because the statistician was not included in the review of the manuscript, I would list his/her contribution in the acknowledgements. Some input from a statistician was needed during the trial developments (power calculations, etc.) but they are not typically involved in the collection of data. Much like Medical Writers, they are integral to the process, but should not typically be listed as authors.* (Publication professional) - *Since the lead clinical investigator believes the statistician contributed substantially to data analysis and interpretation, which is scientific contribution, it merits invitation for authorship.* (Medical writer) |
|  | **Case 3 –** Acknowledging a Medical Writer (n=151) | - *I do not believe it is ethical for professional writers to be authors on peer-reviewed papers. This is a technical function and does not contribute intellectually to reporting of the study.* (Editor) - *Writing is more than putting facts together, choosing the wording is an interpretation of the results.*(Clinical investigator) - *Drafting the manuscript means giving an important contribution to the work. In particular, if the medical writer managed the presentation of the results thanks to his/her scientific knowledge.*(Publication professional) - *I believe medical writers should be listed as authors in those instances where they have contributed from the first draft through to submission. (I recognize that this would require revision of ICMJE guidelines).* (Medical writer) - *The medical writer would write the manuscript based on the report, and would as such not be involved in any scientific contribution to the manuscript – except from the science of writing clearly and readable.*(Medical writer) |
|  | **Case 4 –** Removing an Author (n=11)  [No responses from journal editors] | - *This is important work.*(Clinical investigator) - *As a substantial contributor, it would be wrong to exclude them altogether.* (Publication professional) - *The investigator made a substantial intellectual contribution. i would add his/ her views a s possible variations of interpretation in the discussion section.* (Publication professional) - *Although the investigator disagrees with the data interpretation, he/she did contribute substantially to trial design and should be acknowledged.*(Medical writer) |
|  | **Case 5 –** Contribution from contracted organization (n=199) | - *If the scientist developed a novel assay specifically for this clinical trial, it becomes an important part of the study methodology and should be described in the manuscript and the scientist named as an author.*(Editor) - *Significant scientific contribution to the success of this clinical trial and direct impact on the ability to publish the trial.*(Clinical investigator) - *A clinical trial is fundamentally about the clinical results. No matter how essential the bioassay is to the conduct of the trial, it is still not the main point of the research.*(Publication professional) - *Despite being contracted to conduct the research it seems clear that the CRO scientist provided crucial intellectual contribution to the success of the project which should, ethically, be indicated through authorship.* (Publication professional) |
|  | **Case 6 –** No/Unclear Final Approval (n=106) | - *The investigator needs to be recognized for their efforts but the journal has the chance to ensure agreement with the final version of the manuscript*. (Editor) - *No response = no contribution = no team spirit*(Clinical investigator) - *The clinical investigator contributed substantially to the trial=need to be author. The work needs to be recognized. The fact that the author contributed to the study indicates that he/she is interested in the outcome (results, publication). (*Clinical investigator) - *Just because someone prepares a manuscript does not mean he/she takes all the credit. Credit should be given where credit is due. The focus of the manuscript is the research without which there would be no manuscript.*(Publication professional) - *To list the investigator as an author is much more appropriate way to recognize the contribution than list the name of the investigator in the acknowledgement section with expectation that the formal approval of the submitted manuscript version would come during the review process.* (Medical writer) |
|  | **Case 7 –** No drafting/revising of manuscript (n=174) | - *Based on the clinicians significant contribution, he/she has the right to authorship and this right cannot be taken away, unless in extreme circumstances of scientific misconduct.*(Editor) - *As the clinician has already completed the trial, it is only fair to include his name in the authorship.* (Clinical investigator) - *Again, issues of proprietary information cannot trump intellectual contribution. In this case the sponsoring company will simply have to work to ensure their IP is protected while permitting recognition as coauthor.* (Publication professional) - *The other investigators may feel that the clinician has provided a substantial enough contribution to warrant authorship, and he/she is already aware of the data/results.*(Medical writer) - *Lots of people involved in clinical trials do not become authors of the trial papers. It seems odd for a clinician to insist on involvement despite not being involved in the manuscript.* (Medical writer) |

*The examples provided are representative by respondent group and case categorized into different themes.
